# Effects of Ambient Illuminance on Explicit and Implicit Altruism: The Mediation Roles of Perceived Anonymity and Satisfaction with Light

**DOI:** 10.3390/ijerph192215092

**Published:** 2022-11-16

**Authors:** Taotao Ru, Yongjun Ma, Luojin Zhong, Qingwei Chen, Yiyang Ma, Guofu Zhou

**Affiliations:** 1 National Center for International Research on Green Optoelectronics, South China Normal University, Guangzhou 510006, China; 2 Lab of Light and Physio-Psychological Health, School of Psychology, South China Normal University, Guangzhou 510631, China; 3 School of Professional Development and Research on Primary and Secondary Education, South China Normal University, Guangzhou 510631, China; 4 Guangdong Provincial Key Laboratory of Optical Information Materials and Technology & Institute of Electronic Paper Displays, South China Academy of Advanced Optoelectronics, South China Normal University, Guangzhou 510006, China

**Keywords:** light, implicit association test, dictator game, self-control, anonymity, satisfaction with light, altruism

## Abstract

Ambient light plays a key role in social interactions, and the effects of ambient light on explicit altruism have been widely documented. However, whether ambient light affects implicit altruism and the potential mechanisms underlying the effect remain largely unknown. The current study aimed to explore the effects of ambient illuminance on explicit and implicit altruism simultaneously, and to determine the potential mediation role of subjective mood, state self-control perceived anonymity and satisfaction with light. A one-factor (Illuminance: dim (100 lx) vs. bright (1000 lx) at eye level), between-subjects design was employed in the current study, during which seventy-eight undergraduates (52 females, 18–25 years old) were assigned to two groups, with participants in each group undergoing both the dictator game assessing explicit altruism and the implicit association test (IAT) assessing implicit altruism under one of two illuminance conditions. Meanwhile, subjective mood, state self-control, perceived anonymity and satisfaction with light were also assessed with questionnaires at the beginning or/and at the end of the experiment. Results revealed that participants tended to allocate more money in the dictator game and showed a higher state self-control, satisfaction with light and lower perceived anonymity under bright versus dim illuminance condition, whereas the performance in IAT and subjective mood revealed no statistically significant effects of illuminance. The promoting effect of bright illuminance on explicit altruism was partially mediated by perceived anonymity and satisfaction with light, but not by state self-control. These findings suggest that ambient light holds the potential to regulate psychological well-being and thus facilitate prosocial behavior, but such benefits are dependent on the type of task.

## 1. Introduction

Light, as a fundamental dimension of the physical environment, acts as a vital contributor affecting human behavior and health [1,2,3]. In addition to light’s effects on image forming (IF) function, a growing number of studies have been conducted to explore the non-image forming (NIF) effects of light during recent decades [4,5], among which the effects of light on alertness [6], affective state [7], cognitive performance [8], sleep and circadian rhythms [9] were widely reported. In addition to the effects of ambient light on psychological and biological functioning, the associations between ambient light and social behavior attributes have also been investigated in a few field and laboratory studies [10,11,12,13,14,15,16,17,18,19,20]. Among the limited available evidences, the effects of bright versus dim illuminance on altruism have attracted more attention in the field of social and environmental psychology.

Altruism is a form of prosocial motivation that benefits others while being costly to oneself [21]. According to the notion of dual-processing accounts, altruism can be expressed as explicit altruism and implicit altruism [22]. Explicit altruism is consciously controlled and requires cognitive resources to process, while implicit altruism is processed in an automatic and unconscious manner [22]. Explicit altruistic behavior can be measured by self-rating questionnaires (such as the Self-Report Altruism scale [23]) or behavioral tasks (such as the dictator game [24]). Implicit altruistic attitudes can be captured by implicit measurements, such as the classical Implicit Association Test [25,26].

As a type of social animal, social interactions are ubiquitous and of vital importance for human beings. A recent meta-analysis [27] suggested that altruism is significantly associated with well-being (including psychological functioning and physical health) and the pooled effect size is modest (r = 0.13). Hui further proposed that a positive feedback loop might exist between altruism and well-being [28]. Thus, if altruism-promoting lighting scenarios were created, they might hold great potentials to improve social well-being, which would fulfill the requirements of integrative lighting [3] in the future.

Field-based studies have consistently revealed the positive association between the intensity of daylight and altruistic behaviors. For example, Cunningham [13] found that sunshine could promote helping behavior. In a following study, Guéguen and Lamy [14] reported that participants were more prone to offer kind statements to strangers on sunny days when compared to cloudy days. These findings provide evidences for the notion of the “Sunshine Samaritan” [13].

Meanwhile, a few researchers have made efforts to investigate the effects of artificial light on altruism in a well-controlled laboratory environment. The findings, however, revealed rather inconsistent effects of bright versus dim illuminance on explicit altruistic outcomes. Some studies reported the promotional effects of bright illuminance on altruism. For instance, brightness induced by either artificial bright versus dim illuminance or by wearing clear glasses versus sunglasses significantly reduced self-interested performance in the dictator game, and the effects were robust [15]. Similarly, Chiou and Cheng [16] reported that participants tended to share more money with the recipient under bright versus dim illuminance condition while performing a one-shot, anonymous version of the dictator game. Participants under bright versus dim illuminance condition (500 lx vs. 30 lx) tended to believe that the dictator would treat them unfairly and allocated less money to the recipient in the dictator game [17].

On the contrary, other studies indicated that dim versus bright illuminance could promote altruism. By employing a 2 (Illuminance: 150 lx, 1500 lx) × 2 (Correlated Color Temperature: 3000 K, 4200 K) design, Baron et al. [18] found that participants under the dim warm light condition (150 lx, 3000 K) were more willing to devote their time and money to helping others and were more likely to use cooperative means to cope with interpersonal conflicts when compared to three other light conditions. A following study found a dim-illuminance-induced improvement in conflict resolution [19]. Similarly, Steidle et al. [20] found that dark versus bright illuminance could promote cooperation. Besides these empirical studies, one light therapy study using ecological momentary assessment reported that exposure to a relatively bright illuminance (10,000 lx) in the morning improved mood but increased quarrelsomeness and decreased submissiveness [12].

In summary, field-based studies have evidenced correlations between daylight and altruism, rather than purely causal effects. The studies manipulating artificial light in a well-controlled laboratory provide—although not consistent—evidences for the causal effects of light on altruism. These inconsistent findings from laboratory studies might indicate the fact that the altruism outcomes may not be a direct consequence of the light manipulation; instead, they may be due to the light-induced regulation of other candidate variables. The previous studies reported four potential psychological variables (mood, perceived anonymity, state self-control and satisfaction with light) that occasionally linked ambient light with prosocial behavior attributes [13,15,18,19,29,30]. To date, the potential mechanism underlying the relationship between ambient light and altruism has not been determined, but exploring the potential mediators could also increase our knowledge of the underlying mechanism.

Prosocial behavior is profoundly affected by positive affective experiences (such as happiness, gratitude and pride) or negative affective experiences (such as shame, guilt and sadness) (see [31] for a review). Exposure to short-term light could significantly regulate the affective state [32,33]. Hence, mood was expected to be a potential mediator in the association between light and altruism, which was firstly claimed by Cunningham [13] to explain the notion of the “Sunshine Samaritan”. In a following study, Baron et al. [18] found that participants under dim versus bright illuminance (150 lx vs. 1500 lx) were willing to donate more time as unpaid volunteers, and the authors supposed that this effect might be mediated by the dim-illuminance-induced positive mood. This assumption has not been proposed in studies by Chiou and Cheng [16] and Kombeiz et al. [19]. Specifically, Chiou and Cheng reported no significant effect of illuminance on mood, and subjective mood did not correlate with volunteering willingness [16]. In study by Kombeiz et al., both positive and negative mood were taken as control variables in exploring the effects of bright versus dim illuminance on conflict resolution [19]. Thus, empirical evidences for the mediation role of mood are still required.

In addition, perceived anonymity was proposed to be another potential mediator in the association between light and altruism [15]. Identity is concealed or blurred under darkness or dim illuminance condition. This dim-illuminance-induced sense of illusory anonymity could provide an opportunity for moral transgressions, criminal acts and self-interested behavior as well [34]. Zhong et al. [15] firstly proposed this hypothesis and tested it in an empirical study. Their findings revealed that bright illuminance inhibited self-interested behavior in the dictator game, and the mediation analysis revealed that the perceived anonymity functioned as a mediator between light and altruism. In contrast, null effects of illuminance on perceived anonymity were revealed in the following studies [35,36].

Previous studies have also shown that individuals with high self-control ability were inclined to act in a more other-interested manner than those with low self-control ability [37,38], and this pattern was reversed when faced with crisis situations [39]. The effects of ambient light on state self-control were occasionally reported in previous studies [29,35,40,41,42]. For example, exposure to bright versus dim illuminance (1500 lx vs. 150 lx) could increase participants’ public self-awareness and lead to controlled self-regulation [35]. Kang et al. [29] further reported that both warm bright light (4000 K, 1000 lx) and cool dim light (6000 K, 100 lx) improved participants’ self-control. However, whether state self-control plays a mediating role in the effect of ambient light on altruism still remains an open question.

Satisfaction with light was recently proposed as a contributor to light’s regulation of social perception in study by Kombeiz et al. [19]. This assumption was tested in their follow-up study exploring the effects of different light conditions (cool bright light (5500 K, 1500 lx), cool dim light (5500 K, 150 lx), warm bright light (2500 K, 1500 lx), warm dim light (2500 K, 150 lx)) on social perceptions of a stranger [30]. The results revealed that satisfaction with light was positively associated with social perceptions of warmth and competence. Given these findings, satisfaction with light might be another mediator linking light and social behavioral attributes.

As mentioned above, altruism could be expressed as explicit altruism and implicit altruism. However, the current studies exploring the effects of ambient light on altruism commonly employ tasks to assess explicit altruism, and the effects of ambient light on implicit altruism—to the best of our knowledge—have been scarcely investigated. Previous studies have also established that significant discrepancies exist between explicit and implicit measures [43], and altruism is no exception. For example, no significant association between implicit altruistic attitudes and explicit altruistic behavior was revealed [25]. In addition, explicit altruism might be susceptible to the social desirability effect due to the nature of explicit measurements, while implicit altruism is immune to this effect. Thus, to fill this gap, the first aim of the current study was to investigate the effects of ambient bright versus dim illuminance on explicit altruistic behavior, as assessed with the dictator game, and implicit altruistic attitude as assessed with the implicit association test (IAT), simultaneously. Moreover, the mechanisms underlying the effects of ambient light on altruism remain controversial and empirical evidences for the reported potential mediators are still lacking. Therefore, the second purpose of the current study was to explore the potential mediating role of subjective mood, perceived anonymity, state self-control and satisfaction with light in the association between ambient illuminance and altruism by employing a mediation analysis approach.

## 2. Materials and Methods

### 2.1. Design

The study employed a one-factor (Illuminance: bright vs. dim), between-subjects design. The light of 100 lx at 4000 K and 1000 lx at 4000 K at eye level (approximately 120 cm above the ground) was employed in the bright illuminance condition and dim illuminance condition, respectively. Each participant visited the laboratory once and was randomly assigned to either the bright illuminance condition or dim illuminance condition, during which both the dictator game task and the implicit association test were administrated. The order of the two tasks was counterbalanced across participants.

### 2.2. Participants

Seventy-eight participants (52 females, *M_age_* = 20.87 years, *SD* = 1.79) were recruited via online advertisements at local universities. Half of the participants were assigned to the bright illuminance condition (39 participants, 27 females, 20.92 ± 1.90 years old) and the remaining half were assigned to the dim illuminance condition (39 participants, 25 females, 20.82 ± 1.70 years old). The demographics of participants were balanced between two illuminance conditions (for age: *t* =0.25, *p* = 0.80; for gender ratio: χ^2^ = 0.23, *p* = 0.63).

All employed participants were right-handed and had normal or corrected-to-normal vision. They were instructed that drinks and food containing caffeine and alcohol were forbidden on the experimental day and the day before the formal experiment. They reported no concerns regarding anxiety symptoms (score of Self-rating Anxiety Scale < 50 [44]) and had no sleep complaints regarding the preceding night (score of Groningen Sleep Quality Scale < 5 [45]). The study was approved by the Ethical Committee of South China Normal University (Approval No.: SCNU-PSY-2020-1-044). All participants gave their written informed consent and were paid for their participation.

### 2.3. Experimental Scenario

**Figure 1:**
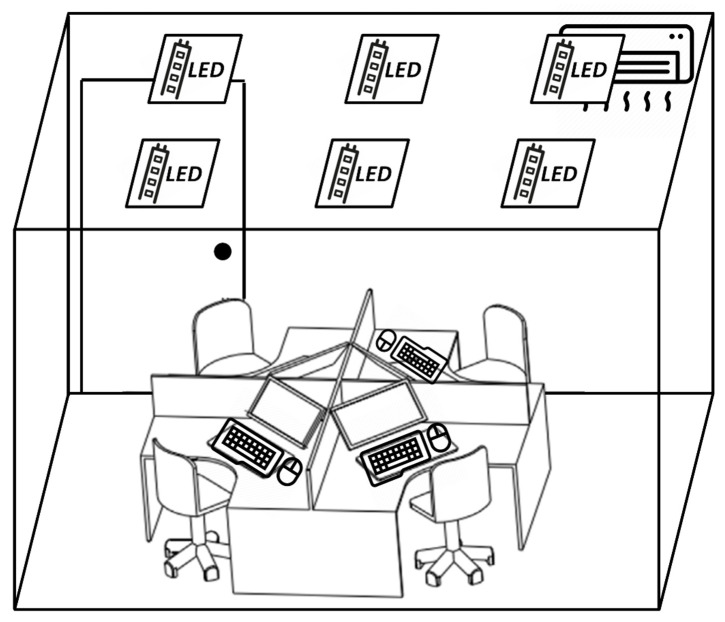
The layout of the laboratory. Note that only one participant was scheduled at a time.

The experiment was conducted in an intelligent lighting laboratory, which was a soundproof and windowless room (3.6 m × 3.6 m × 2.8 m) with off-white walls and ceilings and a dark grey floor (with 90.12%, 90.12% and 18.88% reflectance, respectively). Four separate workstations with one light grey table (1.2 m × 0.8 m, 50.77% reflectance) and one black chair were created. One white all-in-one computer (Lenovo C260, 19.5in, Lenovo, Beijing, China) with a keyboard and a mouse was placed on each table. Six tunable white luminaires (Philips RC099V, Philips, Foshan, China) were mounted on the ceiling of the room. Each luminaire (0.6 m × 0.6 m) had a translucent cover with an integrated diffuser. The layout of the laboratory is shown in Figure 1.

**Figure 2:**
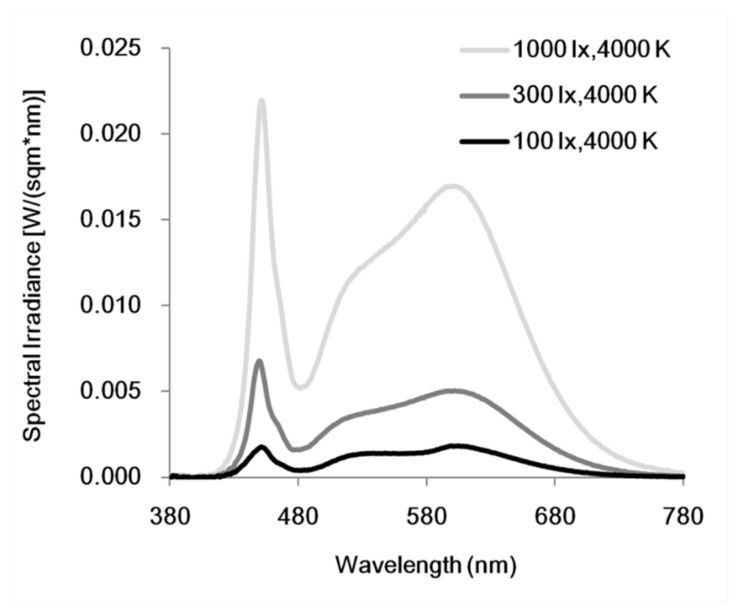
Spectral power distributions measured at eye level for three light conditions employed in the current study (1000 lx for bright illuminance condition; 100 lx for dim illuminance condition; and 300 lx for light adaption).

The basic indoor lighting and experimental light settings were provided by six luminaires, and the illuminance of ambient light was measured vertically at eye level (approximately 120 cm above the ground) prior to the formal laboratory study using a calibrated spectroradiometer (JETI Specbos1202, JETI Technische Instrumente GmbH, Jena, Germany). The height of the chair was adjusted individually to reach the same eye height. The spectral power distributions for light adaption and the two experimental light conditions are displayed in Figure 2. The indoor temperature was kept at 26 °C using an air conditioner.

### 2.4. Measurements

#### 2.4.1. Mood

Subjective mood was evaluated using the Positive and Negative Affect Schedule (PANAS) [46], which includes ten positive and ten negative adjectives to measure positive mood (α = 0.866) and negative mood (α = 0.911), respectively. Participants responded on a 5-point Likert scale ranging from “1” (extremely slight) to “5” (extremely strong).

#### 2.4.2. Self-Control

State self-control was assessed with the 5-Item-Skala zur Messung der momentan verfügbaren Selbstkontrollkapazität (SMS-5) [47], which is a brief version used for measuring the currently available self-control capacity, developed from the 25-item State Self-Control Capacity Scale (SSCCS) [48]. The participants were instructed to rate their state self-control on a 5-point Likert scale (1 “completely inaccurate”, 3 “neither inaccurate, nor true”, 5 “completely true”). A high score indicates a higher state of self-control. The Cronbach’s alpha for SMS-5 in the current study was 0.829.

#### 2.4.3. Perceived Anonymity

Perceived anonymity was evaluated by five 5-point Likert-scale items adopted from a study by Zhong et al. [15] (1 “strongly disagree”, 3 “indeterminacy”, 5 “strongly disagree”). A high score indicates a high level of perceived anonymity (α = 0.693 in the current study).

#### 2.4.4. Satisfaction with Light

Satisfaction with light was assessed by seven items retrieved from Van Den Wymelenberg and Inanici [49] using a 5-point Likert scale (1 “very strongly disagree”, 3 “neither agree or disagree”, 5 “very strongly agree”), with a high score indicating a high level of satisfaction with light (α = 0.908 in the current study).

#### 2.4.5. Brightness

To check the validation of the illuminance manipulation in the current study, the perceived brightness of ambient light in the bright and dim illuminance conditions was evaluated by one item retrieved from Flynn et al. [50] that ranged from 1 “very dim” to 5 “very bright”.

#### 2.4.6. Explicit Altruism: Dictator Game

The dictator game is widely used to measure altruistic behavior [15,16]. In the current study, we utilized this task paradigm to measure explicit altruism. The participant was informed that they would play a dictator game together with one unfamiliar participant who sat in another laboratory room. The distributor would be provided 10 RMB virtually and he/she could decide for himself/herself how much to distribute to the recipient in the other room by entering the number in an online program. The rest of the money would be an additional bonus awarded to the distributor. The recipient had no opportunity to reject the distribution. The participants were told that the role of distributor and recipient was determined by ballot. In fact, all participants were assigned as the distributors and the recipients did not actually exist in the current study.

#### 2.4.7. Implicit Altruism: IAT

IAT was used to measure the implicit amplitude towards multiple altruistic behaviors, such as blood donation [51] and organ donor registration [52]. The self versus other interest IAT was recently developed to specifically measure implicit altruism [25,53,54]. The standard seven blocks of IAT, as shown in Table 1, were used in the current study. Block 4 is a compatible block and block 7 is an incompatible block. The employed concept words and attribute words are presented in Table 2, obtained from Jiang et al. [25]. The reaction times for trials in the compatible block and incompatible block were computed.

### 2.5. Procedure

**Figure 3:**
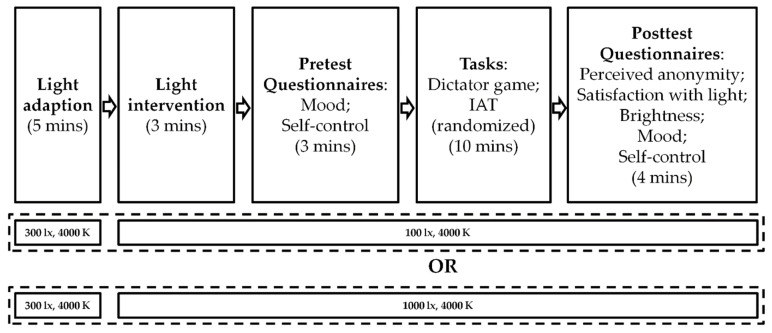
A schematic representation of the overall study protocol.

The overall study protocol is depicted in Figure 3. The qualifying participants were randomly assigned to either the bright illuminance condition or dim illuminance condition upon their arrival at the laboratory. Only one participant was scheduled at a time. The formal experiment always started with a 5 min common “pre-lighting” stage (300 lx at 4000 K at eye level) for light adaption, which has been documented to have an influence on subjective evaluations given to lighting conditions that immediately follow [55,56,57]. After the light adaptation phase, the light was changed either to the dim illuminance or the bright illuminance. The participants were instructed to do nothing for three minutes after the light transition and then fill out the pretest questionnaires to assess their current mood and state self-control. Afterwards, the dictator game and IAT were administrated with a counterbalanced order across participants. At the end of the experiment, the participants were asked to finish the posttest questionnaires to assess their mood, state self-control, perceived anonymity, satisfaction with light and brightness perception. Additionally, all participants were asked whether they had guessed the purpose of the current study, to ensure that the study’s purpose was unknown to participants. To avoid the potential time-of-day variances in the NIF effects of light that were documented in previous studies [58,59], the current study was conducted both during daytime and in the early evening (9:00–21:00), in winter (from December to January), in Guangzhou, China.

### 2.6. Statistical Analysis

For subjective indicators as assessed with questionnaires, the average scores were computed before the formal analysis. For IAT, the reaction times larger than 3000 ms were discarded [25]. For the trials with inaccurate responses, 600 ms was added to the reaction times. The D score, as the indicator of explicit altruism, was calculated with the following equation: D score = (Mean RT_block7_ − Mean RT_block4_)/SD RT_all_ [60]. To examine the difference between bright and dim illuminance conditions, the independent-sample *t*-test was employed for subjective indicators and task performance. To explore the potential mediation role of perceived anonymity, satisfaction with light, mood and self-control, correlation analyses and regression analyses between potential moderators and altruism performance were conducted first and then the PROCESS macro bootstrapping procedure (n = 5000, Model 4) [61] was employed. SPSS 22.0 (IBM, Armonk, NY, USA) was used for all statistical analyses.

## 3. Results

### 3.1. Effects of Illuminance on Subjective Indicators

The descriptive data of subjective indicators and the results of the *t*-test for the differences between the two illuminance conditions on subjective indicators are listed in Table 3. The independent-sample *t*-test for perceived brightness during the posttest session revealed a significant difference between the bright illuminance condition and dim illuminance condition. Participants rated the light brighter under the bright versus dim illuminance condition, which indicated that the manipulation of illuminance was valid in the current study.

The *t*-test for subjective positive mood, negative mood and state self-control measured during the pretest session revealed no significant differences between the bright illuminance condition and dim illuminance condition (all *p* > 0.05). This was also the same for the positive mood and negative mood during the posttest session. However, the state self-control revealed a significant difference between the two illuminance conditions during the posttest session, with participants showing higher state self-control under the bright illuminance condition than those under the dim illuminance condition (*p* = 0.01). Similarly, the subjective satisfaction with light and the perceived anonymity also revealed significant differences between the two illuminance conditions (*p*s < 0.0001), with participants under the bright illuminance condition showing higher satisfaction with light and a perceived higher level of anonymity than those under the dim illuminance condition.

### 3.2. Effects of Illuminance on Altruism

As for explicit altruism, the independent-sample *t*-test revealed a significant difference in the amount of allocated money, with participants under the bright illuminance condition preferring to allocate more money to the recipient than those under the dim illuminance condition (see Table 3 for statistics). In contrast, the D score in IAT revealed no significant difference between the bright illuminance condition and dim illuminance condition, suggesting no statistically significant effect of illuminance on implicit altruistic performance (see Table 3 for statistics).

### 3.3. Mediation Results

Due to the absence of statistically significant effects of the illuminance condition on IAT performance and positive and negative mood, the D score in IAT and mood were not included in the mediation analyses. First, the correlation matrix among state self-control, perceived anonymity, satisfaction with light and the amount of allocated money in the dictator game were computed (see Table 4). Results showed that the amount of allocated money positively correlated with satisfaction with light (*p* < 0.001) and negatively correlated with perceived anonymity (*p* < 0.001), whereas no significant correlation between the amount of allocated money and state self-control was revealed (*p* = 0.24).

**Figure 4:**
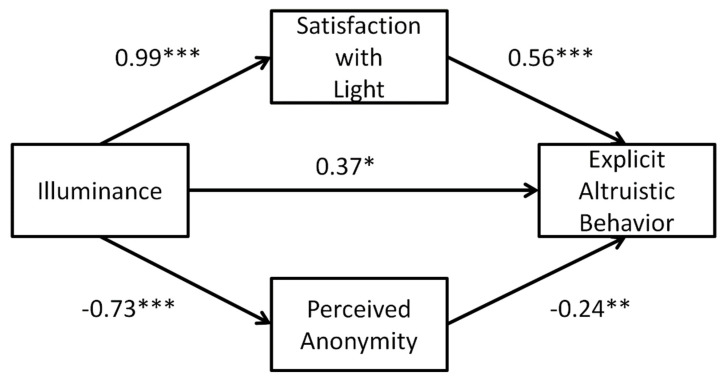
The mediation effects of satisfaction with light and perceived anonymity underlying the effect of illuminance on explicit altruism. Path values are the standardized path coefficients (* *p* < 0.05, ** *p* < 0.01, *** *p* < 0.001).

According to these significant correlations, the stepwise regression analyses were conducted between the illuminance, perceived anonymity, satisfaction with light and the amount of allocated money. The regression results are shown in Table 5 and Figure 4. Results showed that illuminance could significantly predict the amount of allocated money, perceived anonymity and satisfaction with light (*p* < 0.001, *p =* 0.0009 and *p* < 0.001, respectively). In the regression model including all four variables, the results showed that the satisfaction with light (*β* = 0.56, *p* < 0.001) and perceived anonymity (*β* = −0.24, *p* = 0.0039) significantly predicted the amount of allocated money, while the predictive role of illuminance on the amount of allocated money still remained significant (*β* = 0.37, *p* = 0.03).

Furthermore, the PROCESS macro bootstrapping procedure (n = 5000, Model 4) [61] was employed to test whether the effect of illuminance on explicit altruism was mediated by the satisfaction with light or perceived anonymity. As to the mediating effect of satisfaction with light, the 95% bootstrapped confidence interval (CI) for the indirect effect of illuminance on explicit altruism ranged from 0.45 to 1.33. Likewise, the perceived anonymity revealed a significant mediating effect (95% CI: 0.02–0.58). The comparison of the mediation effect of satisfaction with light and perceived anonymity revealed a significant difference (95% CI: 0.08–1.15), suggesting that the mediation effect of satisfaction with light was significantly larger than that of perceived anonymity (see Table 6).

## 4. Discussion

The previous studies exploring the effects of illuminance on altruism particularly focused on explicit altruism and did report inconsistent effects of bright versus dim illuminance on altruistic performance. Meanwhile, the potential pathway by which the ambient illuminance influences altruism is far from conclusive due to the employed differential study paradigms, light manipulations and mediators of interest across different studies. Extending previous studies, the current study was conducted to investigate the effects of ambient illuminance on explicit altruism and implicit altruism simultaneously in a well-controlled laboratory environment. Moreover, the current study was the first time—to the best of our knowledge—to test the potential mediation role of multiple psychological variables, including subjective mood, perceived anonymity, state self-control and satisfaction with light, in the association between ambient illuminance and altruism using a mediation analysis approach. The findings revealed that bright versus dim illuminance promoted explicit altruism, and this effect was partially mediated by bright-illuminance-induced lower perceived anonymity and higher satisfaction with light, but not mediated by bright-illuminance-increased state self-control. Ambient illuminance exerted no statistically significant effects on implicit altruism and subjective mood.

### 4.1. The Effects of Illuminance on Altruism

In the current study, both the dictator game and IAT were employed to explore the effects of ambient illuminance on explicit and implicit altruistic performance. The findings revealed a task-dependent effect of bright versus dim illuminance on altruism. Exposure to bright illuminance (1000 lx at eye level) promoted explicit altruism as the participants under the bright versus dim illuminance condition tended to allocate more money to the unfamiliar recipient in the dictator game, while it had a neglectable effect on implicit altruism as assessed with the D score in IAT. The promoting effect of bright versus dim illuminance on explicit altruism was in line with previous studies reporting that bright versus dim illuminance condition increased the likelihood of prosocial behavior [13,14,15,16]. Interestingly, the current findings revealed that illuminance exerted a null significant effect on implicit altruism, suggesting a task-specific effect of ambient light on altruism.

The absence of a significant effect of bright illuminance on implicit altruism might be partly explained by the fact that implicit altruistic attitude remains relatively stable and resists any change caused by external cues according to the assumption of classical dual-construct theories [43]. In addition to the task’s nature, the limited manipulation of ambient illuminance employed in the current study could also be a potential contributor. Although implicit social cognition remains relatively stable, it is not inevitably unchangeable [43]. Multiple interventions have been developed and a network meta-analysis revealed that changes in implicit measures are still possible, although the effects are weak [63]. Whether a larger range (such as 10 lx and 1500 lx) or multiple illuminance levels (such as 100 lx, 500 lx and 1000 lx) could result in the significant regulation of implicit altruism remains unknown. As an innovative and practical strategy to benefit altruistic attitudes, the study of which light settings could function as an effective intervention to improve implicit altruism warrants further attention in future studies.

### 4.2. The Mediation Role of Perceived Anonymity and Satisfaction with Light

Previous studies have reported that several psychological variables, such as perceived anonymity, state self-control and mood, hold the possibility to mediate the relationship between ambient light and prosocial behavior [15,18,29]. In addition, subjective satisfaction with light was particularly involved as a potential mediator, as it was documented to be correlated with social perception [30]. Among these potential mediators, the current findings revealed that bright versus dim illuminance resulted in lower perceived anonymity, higher state self-control and satisfaction with light, but no significant regulation of subjective positive mood nor negative mood. The finding on the effect of illuminance on perceived anonymity was in line with a previous study reporting that bright illuminance significantly decreased perceived anonymity [15], but contradicted another two studies reporting that bright illuminance induced no significant regulation of perceived anonymity [35,36]. These discrepancies might be due to differences in light manipulation. For instance, 1500 lx vs. 150 lx and 372 lx vs. 40 lx were employed in these two studies [35,36], respectively, while 1000 lx vs. 100 lx was employed in the current study. The effect of illuminance on satisfaction with light was scarcely investigated [19,30], while previous studies had occasionally reported significant effects of bright versus dim illuminance on state self-control [29,35,40,41,42] and mood [16,59,64,65,66]. Again, the differences in light manipulation make it difficult to directly compare the findings of previous studies and the current one. Moreover, the differential questionnaires employed to assess subjective mood and state self-control could also be potential contributors to these inconsistencies. The adjectives in the PANAS employed in the current study might be so intense that they cannot effectively capture the changes in affective state induced by ambient light. More brief and sensitive scales should be developed to effectively trace the dynamics of affective states under various light conditions in future studies, as Itzhacki et al. [67] have suggested.

The mediation analyses revealed that both the perceived anonymity and satisfaction with light partially mediated the association between light and explicit altruism. Meanwhile, the mediation role of satisfaction with light was significantly larger than that of perceived anonymity (0.85 vs. 0.27, 95% CI [0.08, 1.15]). This finding emphasized the vital role of satisfaction with light underlying the effects of ambient light on social behavior attributes. The mediation role of perceived anonymity was also evidenced in a previous study exploring the effect of brightness (manipulated by wearing sunglasses or clear glasses) on explicit altruistic behavior in the dictator game [15]. The current study represents the first time—to our knowledge—that the mediation role of state self-control and satisfaction with light in the association between illuminance and altruism has been investigated. Unfortunately, the mediation role of state self-control was not established. Although the current study provides empirical evidence for the mediation of satisfaction with light, more researches are needed to test whether the light-induced regulation of satisfaction would also function as a predictor of other prosocial behaviors. In addition, considering the fact that satisfaction with light and perceived anonymity did partially mediate the association between ambient illuminance and explicit altruism, other potential mediators need to be explored in future studies.

Some limitations need to be mentioned in the current study. Firstly, a between-subject design was employed in the current study, thus leading to possible confounding effects of individual differences, such as personality. Secondly, in addition to the potential mediators, the previous studies had also suggested that there were potential moderators and group differences, such as self-construal [68] and gender [69], in the association between ambient light and prosocial behaviors. It would be of great value to explore the moderators and mediators within one model, to obtain a more comprehensive picture of the lighting effects on prosocial behavior. Thirdly, the light level was only manipulated to two levels within a small range (i.e., 100 vs. 1000 lx); as discussed above, multiple light levels and larger ranges of contrasting light are necessary in one study paradigm to draw a dose–response curve for the effects of illuminance on altruistic performance. Furthermore, the duration and timing of light exposure might be another seminal feature that warrants further endeavors. The relatively short duration employed in the current investigation might hinder the detection of the delicate effects of light on implicit altruism.

Despite the limitations mentioned, the findings together with the current literature suggesting that exposure to bright indoor illuminance would be a practical and effective strategy to promote altruistic performance. For instance, exposure to brighter light may be of benefit in public interior building spaces or urban roads in the evening, to increase psychological satisfaction and reduce perceived anonymity and thus increase the likelihood of prosocial behavior. In addition, the recommendations for the current interior lighting design mainly aim to maintain circadian rhythms [70,71], while limited attention has been paid to psychological well-being and social behavior. The exploration and creation of optimal lighting conditions for well-being and social behavior should also be a motivation of integrative lighting design.

## 5. Conclusions

Exposure to bright versus dim illuminance increases state self-control and satisfaction with light and decreases perceived anonymity, while subjective positive and negative mood remain unaffected. Ambient illuminance renders a task-specific effect on altruism, with explicit altruistic behavior rather than implicit altruistic outcomes being promoted with bright versus dim illuminance exposure. Satisfaction with light and perceived anonymity partially mediate the association between ambient illuminance and explicit altruism. These findings, together with the current literature, suggest that exposure to bright illuminance holds the possibility to regulate perceived anonymity and satisfaction and thus functions as a practical strategy to promote prosocial behavior.

## Figures and Tables

**Table 1:** Seven blocks of IAT procedure.

Block	No. of Trials	Function	Items Assigned to Left-Key Response	Items Assigned to Right-Key Response
1	20	Practice	Self words	Others words
2	20	Practice	Altruistic words	Non-altruistic words
3	20	Practice	Self words + Altruistic words	Others words + Non-altruistic words
4	40	Test	Self words + Altruistic words	Others words + Non-altruistic words
5	20	Practice	Others words	Self words
6	20	Practice	Others words + Altruistic words	Self words + Non-altruistic words
7	40	Test	Others words + Altruistic words	Self words + Non-altruistic words

**Table 2:** The concept words and attribute words in IAT.

Concept Words		Attribute Words	
Self	Others	Altruistic	Non-Altruistic
自己 (self)	他们 (they)	关爱 (caring)	拒绝 (rejection)
自我 (self)	她们 (they)	帮助 (assistance)	攻击 (aggression)
我们 (we)	别人 (other people)	奉献 (dedication)	藐视 (contempt)
我的 (mine)	外人 (outsiders)	支持 (support)	辱骂 (abuse)
咱们 (we)	他人 (others)	保护 (protection)	欺骗 (chicanery)

**Table 3:** Results of t-tests for the differences between bright illuminance and dim illuminance on subjective feelings and altruism.

	Bright Light	Dim Light	*t*	*p*	Cohen’s *d*
Perceived brightness	4.86 ± 0.96	2.94 ± 1.06	8.35	<0.001	1.89
Positive affect_pretest_	3.21 ± 0.38	3.28 ± 0.46	−0.73	0.47	0.16
Positive affect_posttest_	3.20 ± 0.53	3.21 ± 0.56	−0.12	0.90	0.02
Negative affect_pretest_	1.96 ± 0.55	1.83 ± 0.37	1.29	0.20	0.29
Negative affect_posttest_	1.78 ± 0.72	1.70 ± 0.45	0.55	0.58	0.13
Self-control_pretest_	3.92 ± 0.60	3.86 ± 0.48	0.50	0.62	0.11
Self-control_posttest_	4.01 ± 0.52	3.70 ± 0.54	2.56	0.01	0.58
Satisfaction with light	3.59 ± 0.85	2.65 ± 0.81	4.99	<0.001	1.13
Perceived anonymity	3.27 ± 0.76	3.83 ± 0.69	−3.44	<0.001	0.78
Dictator game (allocated money)	3.36 ± 1.48	1.67 ± 1.11	5.72	<0.001	1.30
IAT (D score)	1.04 ± 0.64	1.29 ± 0.73	−1.61	0.11	0.37

Note: the Cohen’s *d* was calculated to indicate the effect size of the effect of illuminance. Standards of Cohen’s *d*: small: Cohen’s *d* ≤ 0.20; moderate: 0.21 ≤ Cohen’s *d* ≤ 0.49; medium: 0.50 ≤ Cohen’s *d* ≤ 0.79; large: Cohen’s *d* ≥ 0.80) [62].

**Table 4:** Correlations among self-control, perceived anonymity, satisfaction with light and the amount of allocated money in dictator game.

	1	2	3	4
1. Self-control	1			
2. Perceived anonymity	0.09	1		
3. Satisfaction with light	0.28 *	−0.29 *	1	
4. Allocated money	0.14	−0.47 ***	0.72 ***	1

Note: * *p* < 0.05; *** *p* < 0.001.

**Table 5:** Results of regression analyses.

Regression Equation		Fitting Indices		Regression Coefficients	
Outcome Variables	Predictor Variables	R^2^	*F*	*β*	*t*
Allocated money		0.30	32.74 ***		
	Illuminance			0.55	5.72 ***
Satisfaction with light		0.25	24.86 ***		
	Illuminance			0.99	4.99 ***
Perceived anonymity		0.13	11.84 ***		
	Illuminance			−0.73	−3.44 ***
Allocated money		0.61	38.47 ***		
	Illuminance			0.37	2.15 *
	Satisfaction with light			0.56	6.57 ***
	Perceived anonymity			−0.24	−2.98 **

Note: * *p* < 0.05; ** *p* < 0.01, *** *p* < 0.001.

**Table 6:** Results of mediation analyses.

Indirect Effect	Effect Size	Boot SE	Boot 95% CI
Total	1.12	0.25	[0.64, 1.63]
Satisfaction with light	0.85	0.22	[0.45, 1.33]
Perceived anonymity	0.27	0.14	[0.02, 0.58]
Satisfaction with light–Perceived anonymity	0.58	0.27	[0.08, 1.15]

Note: Boot SE: Bootstrap Standard Error; Boot 95% CI: Bootstrap 95% Confidence Interval.

## Data Availability

The raw data supporting the conclusions of this article will be made available by the authors, without undue reservation.

## References

[1] Boyce P.R. (2021). Light, Lighting and Human Health. Light. Res. Technol..

[2] Houser K.W., Boyce P.R., Zeitzer J.M., Herf M. (2021). Human-Centric Lighting: Myth, Magic or Metaphor?. Light. Res. Technol..

[3] Vetter C., Pattison P.M., Houser K., Herf M., Phillips A.J.K., Wright K.P., Skene D.J., Brainard G.C., Boivin D.B., Glickman G. (2022). A Review of Human Physiological Responses to Light: Implications for the Development of Integrative Lighting Solutions. Leukos.

[4] Boyce P.R., Carter D.J. (2018). Lighting Research and Technology: Past, Present and Future. Light. Res. Technol..

[5] Chen Q.W., Ru T.T., Zhai D.G., Huang X.H., Li Y., Qian L., Wang Y.Y., Zhou G.F. (2020). Half a Century of Lighting Research & Technology: A Bibliometric Review. Light. Res. Technol..

[6] Mu Y.-M., Huang X.-D., Zhu S., Hu Z.-F., So K.-F., Ren C.-R., Tao Q. (2022). Alerting Effects of Light in Healthy Individuals: A Systematic Review and Meta-Analysis. Neural Regen. Res..

[7] Do A., Li V.W., Huang S., Michalak E.E., Tam E.M., Chakrabarty T., Yatham L.N., Lam R.W. (2022). Blue-Light Therapy for Seasonal and Non-Seasonal Depression: A Systematic Review and Meta-Analysis of Randomized Controlled Trials. Can. J. Psychiatry.

[8] Siraji M.A., Kalavally V., Schaefer A., Haque S. (2022). Effects of Daytime Electric Light Exposure on Human Alertness and Higher Cognitive Functions: A Systematic Review. Front. Psychol..

[9] Ricketts E.J., Joyce D.S., Rissman A.J., Burgess H.J., Colwell C.S., Lack L.C., Gradisar M. (2022). Electric Lighting, Adolescent Sleep and Circadian Outcomes, and Recommendations for Improving Light Health. Sleep Med. Rev..

[10] Chen Q., Ru T., Zhou J., Li J., Xiong X., Li X., Zhou G. (2018). The Effects of Light on Social Cognition and Social Behavior. Adv. Psychol. Sci..

[11] Xiao H., Cai H., Li X. (2021). Non-Visual Effects of Indoor Light Environment on Humans: A Review. Physiol. Behav..

[12] Hsu Z.Y., Moskowitz D.S., Young S.N. (2014). The Influence of Light Administration on Interpersonal Behavior and Affect in People with Mild to Moderate Seasonality. Prog. Neuro Psychopharmacol. Biol. Psychiatry.

[13] Cunningham M.R. (1979). Weather, Mood, and Helping Behavior: Quasi Experiments with the Sunshine Samaritan. J. Pers. Soc. Psychol..

[14] Guéguen N., Lamy L. (2013). Weather and Helping: Additional Evidence of the Effect of the Sunshine Samaritan. J. Soc. Psychol..

[15] Zhong C.-B., Bohns V.K., Gino F. (2010). Good Lamps Are the Best Police: Darkness Increases Dishonesty and Self-Interested Behavior. Psychol. Sci..

[16] Chiou W.-B., Cheng Y.-Y. (2013). In Broad Daylight, We Trust in God! Brightness, the Salience of Morality, and Ethical Behavior. J. Environ. Psychol..

[17] Yin R., Ye H. (2014). The Black and White Metaphor Representation of Moral Concepts and Its Influence on Moral Cognition. Acta Psychol. Sin..

[18] Baron R.A., Rea M.S., Daniels S.G. (1992). Effects of Indoor Lighting (Illuminance and Spectral Distribution) on the Performance of Cognitive Tasks and Interpersonal Behaviors: The Potential Mediating Role of Positive Affect. Motiv. Emot..

[19] Kombeiz O., Steidle A., Dietl E. (2017). View It in a Different Light: Mediated and Moderated Effects of Dim Warm Light on Collaborative Conflict Resolution. J. Environ. Psychol..

[20] Steidle A., Hanke E.-V., Werth L. (2013). In the Dark We Cooperate: The Situated Nature of Procedural Embodiment. Soc. Cogn..

[21] Kinnunen S., Windmann S. (2013). Dual-Processing Altruism. Front. Psychol..

[22] Evans J.S.B.T. (2008). Dual-Processing Accounts of Reasoning, Judgment, and Social Cognition. Annu. Rev. Psychol..

[23] Philippe Rushton J., Chrisjohn R.D., Cynthia Fekken G. (1981). The Altruistic Personality and the Self-Report Altruism Scale. Pers. Individ. Dif..

[24] Yang C., Wang Y., Wang Y., Zhang X., Liu Y., Chen H. (2020). The Effect of Sense of Community Responsibility on Residents’ Altruistic Behavior: Evidence from the Dictator Game. Int. J. Environ. Res. Public Health.

[25] Jiang D., Wang X.R., Fu L., Zhou R.L. (2008). A Study on Implicit Altruistic Behavior. J. Psychol. Sci..

[26] Perugini M., Conner M., O’Gorman R. (2011). Automatic Activation of Individual Differences: A Test of the Gatekeeper Model in the Domain of Spontaneous Helping. Eur. J. Pers..

[27] Hui B.P.H., Ng J.C.K., Berzaghi E., Cunningham-Amos L.A., Kogan A. (2020). Rewards of Kindness? A Meta-Analysis of the Link between Prosociality and Well-Being. Psychol. Bull..

[28] Hui B.P.H. (2022). Prosocial Behavior and Well-Being: Shifting from the ‘Chicken and Egg’ to Positive Feedback Loop. Curr. Opin. Psychol..

[29] Kang S.Y., Youn N., Yoon H.C. (2019). The Self-Regulatory Power of Environmental Lighting: The Effect of Illuminance and Correlated Color Temperature. J. Environ. Psychol..

[30] Kombeiz O., Dietl E. (2019). Light as a Positive Situational Cue at Work: Satisfaction with Light Relates to Judgements of Other’s Warmth and Competence. Ergonomics.

[31] van Kleef G.A., Lelieveld G.-J. (2022). Moving the Self and Others to Do Good: The Emotional Underpinnings of Prosocial Behavior. Curr. Opin. Psychol..

[32] Li Y., Ru T., Li S., Chen H., Xie S., Zhou G. (2022). Effects of Ambient Light on Mood and Its Mechanism. Adv. Psychol. Sci..

[33] Kong Z., Liu Q., Li X., Hou K., Xing Q. (2022). Indoor Lighting Effects on Subjective Impressions and Mood States: A Critical Review. Build. Environ..

[34] Hirsh J.B., Galinsky A.D., Zhong C.-B. (2011). Drunk, Powerful, and in the Dark: How General Processes of Disinhibition Produce Both Prosocial and Antisocial Behavior. Perspect. Psychol. Sci..

[35] Steidle A., Werth L. (2014). In the Spotlight: Brightness Increases Self-Awareness and Reflective Self-Regulation. J. Environ. Psychol..

[36] Mehta V., Mukherjee S., Manjaly J.A. (2017). Can Lighting Influence Self-Disclosure?. Front. Psychol..

[37] DeWall C.N., Baumeister R.F., Gailliot M.T., Maner J.K. (2008). Depletion Makes the Heart Grow Less Helpful: Helping as a Function of Self-Regulatory Energy and Genetic Relatedness. Personal. Soc. Psychol. Bull..

[38] Balliet D., Joireman J. (2010). Ego Depletion Reduces Proselfs’ Concern with the Well-Being of Others. Gr. Process. Intergr. Relat..

[39] Wang Y., Zhang X., Li J., Xie X. (2019). Light in Darkness: Low Self-Control Promotes Altruism in Crises. Basic Appl. Soc. Psych..

[40] Smolders K.C.H.J., de Kort Y.A.W. (2014). Bright Light and Mental Fatigue: Effects on Alertness, Vitality, Performance and Physiological Arousal. J. Environ. Psychol..

[41] Qian L., Ru T., Chen Q., Li Y., Zhou Y., Zhou G. (2021). Effects of Bright Light and an Afternoon Nap on Task Performance Depend on the Cognitive Domain. J. Sleep Res..

[42] de Vries A., Souman J.L., de Kort Y.A.W. (2020). Teasing Apart Office Illumination: Isolating the Effects of Task Illuminance on Office Workers. Light. Res. Technol..

[43] Greenwald A.G., Lai C.K. (2020). Implicit Social Cognition. Annu. Rev. Psychol..

[44] Wang Z.Y., Chi Y. (1984). Chinese Version of Zung’s Self-Rating Anxiety Scale. J. Shanghai Psychiatry.

[45] Jafarian S., Gorouhi F., Taghva A., Lotfi J. (2008). High-Altitude Sleep Disturbance: Results of the Groningen Sleep Quality Questionnaire Survey. Sleep Med..

[46] Huang L., Yang T.Z., Ji Z.M. (2003). Applicability of the Positive and Negative Affect Scale in Chinese. Chin. Ment. Health J..

[47] Lindner C., Lindner M.A., Retelsdorf J. (2019). Die 5-Item-Skala Zur Messung Der Momentan Verfügbaren Selbstkontrollkapazität (SMS-5) Im Lern- Und Leistungskontext. Diagnostica.

[48] Ciarocco N., Twenge J.M., Muraven M., Tice D.M. (2007). The State Self-Control Capacity Scale: Reliability, Validity, and Correlations with Physical and Psychological Stress.

[49] Van Den Wymelenberg K., Inanici M. (2016). Evaluating a New Suite of Luminance-Based Design Metrics for Predicting Human Visual Comfort in Offices with Daylight. Leukos.

[50] Flynn J.E., Spencer T.J., Martyniuk O., Hendrick C. (1973). Interim Study of Procedures for Investigating the Effect of Light on Impression and Behavior. J. Illum. Eng. Soc..

[51] Warfel R.M., France C.R., France J.L. (2012). Application of Implicit Attitude Measures to the Blood Donation Context. Transfusion.

[52] Joshi M.S., Stevens C. (2017). Implicit Attitudes to Organ Donor Registration: Altruism and Distaste. Health Psychol. Behav. Med..

[53] Marvel J.D., Resh W.D. (2019). An Unconscious Drive to Help Others? Using the Implicit Association Test to Measure Prosocial Motivation. Int. Public Manag. J..

[54] Thornton E.M., Aknin L.B. (2020). Assessing the Validity of the Self versus Other Interest Implicit Association Test. PLoS ONE.

[55] Fotios S., Kent M. (2021). Measuring Discomfort from Glare: Recommendations for Good Practice. Leukos.

[56] Houser K.W., Tiller D.K. (2003). Measuring the Subjective Response to Interior Lighting: Paired Comparisons and Semantic Differential Scaling. Light. Res. Technol..

[57] Tiller D.K., Rea M.S. (1992). Semantic Differential Scaling: Prospects in Lighting Research. Light. Res. Technol..

[58] Rüger M., Gordijn M.C.M., Beersma D.G.M., de Vries B., Daan S. (2006). Time-of-Day-Dependent Effects of Bright Light Exposure on Human Psychophysiology: Comparison of Daytime and Nighttime Exposure. Am. J. Physiol. Integr. Comp. Physiol..

[59] Smolders K.C.H.J., de Kort Y.A.W., Cluitmans P.J.M. (2012). A Higher Illuminance Induces Alertness Even during Office Hours: Findings on Subjective Measures, Task Performance and Heart Rate Measures. Physiol. Behav..

[60] Greenwald A.G., Nosek B.A., Banaji M.R. (2003). Understanding and Using the Implicit Association Test: I. An Improved Scoring Algorithm. J. Pers. Soc. Psychol..

[61] Hayes A.F. (2017). Introduction to Mediation, Moderation, and Conditional Process Analysis: A Regression-Based Approach.

[62] Cohen J. (1992). A Power Primer. Psychol. Bull..

[63] Forscher P.S., Lai C.K., Axt J.R., Ebersole C.R., Herman M., Devine P.G., Nosek B.A. (2019). A Meta-Analysis of Procedures to Change Implicit Measures. J. Pers. Soc. Psychol..

[64] Li Y., Ru T., Chen Q., Qian L., Luo X., Zhou G. (2021). Effects of Illuminance and Correlated Color Temperature of Indoor Light on Emotion Perception. Sci. Rep..

[65] Ru T., Smolders K.C.H.J., Chen Q., Zhou G., de Kort Y.A.W. (2021). Diurnal Effects of Illuminance on Performance: Exploring the Moderating Role of Cognitive Domain and Task Difficulty. Light. Res. Technol..

[66] Ru T., de Kort Y.A.W., Smolders K.C.H.J., Chen Q., Zhou G. (2019). Non-Image Forming Effects of Illuminance and Correlated Color Temperature of Office Light on Alertness, Mood, and Performance across Cognitive Domains. Build. Environ..

[67] Itzhacki J., te Lindert B.H.W., van der Meijden W.P., Kringelbach M.L., Mendoza J., Van Someren E.J.W. (2019). Environmental Light and Time of Day Modulate Subjective Liking and Wanting. Emotion.

[68] Esteky S., Wooten D.B., Bos M.W. (2020). Illuminating Illumination: Understanding the Influence of Ambient Lighting on Prosocial Behaviors. J. Environ. Psychol..

[69] Liu G., Niu X., Lin L. (2018). Gender Moderates the Effect of Darkness on Ethical Behaviors: An Explanation of Disinhibition. Pers. Individ. Dif..

[70] Brown T.M., Brainard G.C., Cajochen C., Czeisler C.A., Hanifin J.P., Lockley S.W., Lucas R.J., Münch M., O’Hagan J.B., Peirson S.N. (2022). Recommendations for Daytime, Evening, and Nighttime Indoor Light Exposure to Best Support Physiology, Sleep, and Wakefulness in Healthy Adults. PLOS Biol..

[71] Schlangen L.J.M., Belgers S., Cuijpers R.H., Zandi B., Heynderickx I. (2022). Correspondence: Designing and Specifying Light for Melatonin Suppression, Non-Visual Responses and Integrative Lighting Solutions-Establishing a Proper Bright Day, Dim Night Metrology. Light. Res. Technol..

